# The biomedical securitization of global health

**DOI:** 10.1186/s12992-023-00915-y

**Published:** 2023-03-04

**Authors:** Jens Holst, Remco van de Pas

**Affiliations:** 1 Department of Health Sciences, Fulda University of Applied Sciences, Leipziger Strasse 123, 36037 Fulda, Germany; 2 Department of Public Health, Lecturer Global Health Policy, Institute of Tropical Medicine, Antwerp, Nationalestraat 155, B-2000 Antwerp, Belgium; 3 Centre for Planetary Health Policy, German Alliance Climate Change and Health, Berlin, Germany

**Keywords:** Global health, Security, Securitization, Globalization, Inequality, Biomedical reductionism

## Abstract

**Background:**

The COVID-19 outbreak has shifted the course in the global health debate further towards health security and biomedical issues. Even though global health had already played a growing role in the international policy agenda, the pandemic strongly reinforced the interest of the media, the general public and the community in cross-border infectious diseases. This led to a strengthening of the already dominant biomedical understanding of global health and the securitization of health in foreign policy.

**Methods:**

This paper critically provides a narrative, iterative review of the health security literature available to date, with a special focus on the development of the currently prevailing concept of health security and the dual trend towards the securitization and biomedicalization of global health.

**Findings:**

In a world increasingly determined by power asymmetries, unequal distribution of opportunities and resources, and inadequate governance structures, securitizing health has become a key feature of global governance. Health security is predominantly based on a concept that neglects the global burden of disease determined by non-communicable conditions rather than by infectious diseases. Moreover, it exhibits a trend towards biomedical solutions and neglects root causes of global health crises.

**Conclusions:**

As important as health security is, the underlying concept driven by biomedical and technocratic reductionism falls short. It widely neglects the social, economic, political, commercial and environmental determination of health. Beyond improved health care and prevention, health-in-all policies are ultimately required for ensuring health security and reducing one of its main challenges, health inequalities within and between countries. Global health security must first and foremost seek to guarantee the universal right to health and therefore emphasise the social, economic, commercial and political determination of health.

## Introduction

The COVID-19 outbreak has highlighted the continuing menace to humanity of global health issues that had been suppressed and considered under control, at least in the Global North. The massive warfare activities in Ukraine in the third year of the pandemic have reawakened the sense of threat particularly in Europe. Both the pandemic and the war, as well as the escalating climate crisis, have made global insecurity more tangible. The existing and likely crises caused by these unfortunate occurrences – mass displacement, increasing prices for products of basic needs such as food and energy, and the overall severe impact on global economy—will burden people in low-income countries most but not at all exclusively [1]. Especially the fatal effects on future generations [2] understandably reinforce the desire for a safer world. Enhanced security is needed in times of growing uncertainty, threatened ancestral rights and dwindling opportunities for a large part of mankind. The current global order has therefore been aptly described as a world risk society [3].

Even if this need for security is comprehensible, the nature of the security discourse in global health and beyond raises fundamental questions and concerns. Instead of pursuing common objectives, such as social stability, global public goods, equity or social justice, and recognizing shared responsibilities by governments across national boundaries, security policies predominantly envisage biomedical threats such as infectious diseases, more or less openly admitting that the focus is on the autonomy of nation states and safeguarding the existing status quo in international relations, however unfair they may be [4]. The utopian exuberance that led to the founding of the WHO and other United Nations (UN) organizations is thus giving way to a pragmatic, predominantly biomedical realism that undermines rather than promotes people’s rights and legal entitlements, as enshrined in the Universal Declaration of Human Rights and in the WHO constitution [5].

The SARS-CoV-2 pandemic has highlighted the extent to which global and public health has become dominated by biomedical approaches. Politicians, policymakers and the media provided virologists and epidemiologists a paramount role in dealing with the pandemic. For a considerable time, they provided the main evidence base for partly far-reaching measures and restrictions. The strong, near-exclusive focus on biomedical sciences and their assessment of the pandemic situation led to the initial ignorance of the non-medical, social determination of health. In Germany, for example, it took the dominant COVID-19 experts and political decisionmakers 14 months to even notice the pronounced socio-economic differences in the burden of the coronavirus infection [6].

The ongoing debate about the COVID-19 pandemic and its interrelation with health security does not sufficiently put the spotlight on the structural causes of global health crises. Despite overwhelming evidence of the social determination of health, political leaders, the media and an influential part of global health actors have focused first and foremost on biomedical approaches [7], while often underestimating or even ignoring the close relationship between socioeconomic status, living conditions and unhealthy lifestyles on the one hand, and both the burden of non-communicable diseases and the severity and lethality of COVID-19 infections on the other [8, 9].

A narrative, iterative review of the available literature on health security indicates an increasing biomedicalization of public health that has contributed significantly to making the linkage between health and security the priority discourse in global health and its governance. Security aspects and considerations have gained both acceptance and importance in the last decades. The quest for security is understandable in an increasingly inequitable, unstable and disjointed world, particularly vis-à-vis the militarization of conflicts and international relations. But a closer look at the concept of health security makes things appear more complicated. It often remains unclear what is meant by this form of security, who defines it and who is being targeted by security policies as well as how health security is to be generated [10]. Health security remains centred on the security of nation states and defence against international threats, rather than prioritizing people’s health and its determination [11]. Policymakers in high-income countries tend to emphasise protection of their populations particularly against external threats, for example bioterrorism, zoonoses and pandemics, whereas many—but not all—global health professionals understand the term in a broader context of social health protection, population health and its determination [12].

Over the last two decades, global health security has often become virtually and almost interchangeably synonymous with global health [13]. However, this perspective rather exhibits a conceptual narrowing than epistemic clarity. Global health security is actually but one area of the global health domain, as it primarily addresses cross-border health threats but not the whole span of global health policy [14, 15]. Meanwhile, a universal definition of global health security is still lacking, despite the abundance of available literature on human and (global) health security. The recent attempt of the WHO, as the United Nations (UN) agency specialised on health, to define global health security falls short as it focuses predominantly on the international spread of acute infectious threats [16]. The fact that the use of the term “health security” by different actors is as widespread as it is inconsistent is by no means attributable to the difficulty of achieving conceptual clarification, but rather to the widely divergent perceptions, interests, priorities, politics, and power relations that exist in the broader field of global health [17]. Despite its widespread use, the World Health Assembly has not yet agreed on a resolution on the concept of ‘health security’, indicating that its scope is contested among member states [18].

## Health security in international foreign policy

Already before the COVID-19 outbreak, global health was high on the international political agenda and played an important role at summit meetings of international fora of high-income and emerging countries such as the “Group of 7” (G7), and the “Group of 20” (G20) [19]. However, the prevailing global health security approach tends to be insufficient for adequately catering to the complexity of challenges. There is a need to harmonise the contemporary security debate with other fundamental health-policy challenges from an equity perspective [20, 21]. Current global health programmes often fail to fulfil the claim to universalism implicitly associated with the term “global” and tend to neglect the requirements of a comprehensive transdisciplinary and interdisciplinary understanding of health security policies. There is still a large discrepancy between the current state of knowledge on improving health security, equity and actual global health politics and practices [22]. This trend is partly attributable to the unexpected resurgence of epidemic risks that were considered defeated or at least controllable in high-income countries in recent years.

Since the last two decades, a rapid (re)-emergence of pathogenic infectious diseases has been seen in several regions of the world, including, but not restricted to, diseases such as SARS, avian influenza, Ebola, Zika, and most recently, COVID-19. These outbreaks have each led to the application of control measures around the world and strongly influenced the global health debate in which securitization has become a prevalent and common theme among global health scholars and practitioners [23]. At the same time, other health threats such as tuberculosis, malaria, or chronic conditions like cardiovascular diseases and diabetes mellitus persisted. However, neither poverty-related infections nor the growing prevalence of non-communicable diseases have ever received the attention they deserve due to the continuous upward trend, particularly in countries in transition [24].

Notwithstanding this alarming global burden of disease trend both in the Global North and South[25], it is not the major killers, but the rapid succession of transnational ‘health crises’ caused by viral infections that repeatedly provoke a state of alert and make the headlines. However, public interest in the general health-related challenges of other countries and continents is usually short-lived and transient. In low-income countries the situation is fundamentally different. Certain health hazards persist and the risk of ‘endemic’ diseases and their underlying ‘syndemics’ is part of everyday life [26]. In the course of epidemiological transition, the disease spectrum is increasingly expanding or even shifting from infectious to non-communicable, chronic diseases. During the last decades, societies in the Global South and elsewhere have been facing a double burden of disease caused by bacterial, viral or other pathogens on the one hand, and health problems commonly referred to as non-communicable diseases (NCD’s) on the other [27]. The simultaneous coexistence of undernourishment, malnutrition or dietary overweight exacerbates the situation [28].

## Securitization of global health

Global health has become an important area of foreign and security policy and hence of international diplomacy [29]. In fact, the UN Security Council has addressed health beyond the issue of epidemics and pandemics and taken the role of a forum to debate and negotiate global health issues; this reflects primarily the political interests of the permanent five, particularly the USA [30]. Health security often shapes the global health debate more than other health issues. Security is frequently encountered as a contextual framework in political health and foreign-policy documents, and the securitization of health has for some time now been considered a key feature in global health governance [31]. Security practices and medical knowledge share a common evolutionary history; to consider and analyse them as independent of each other would be to ignore the historical formation of nation states and also the colonial roots of global health [32].

Security is one of several global health “frames” that are by no means exclusive, but have been driven in recent decades by a deep core of neoliberal values [33]. This implies that in contemporary global health governance there is an overemphasis on agency, while deeper structures, including its embedded ideas and configurations of powers, are neglected [31]. As such, neoliberalism has become the globally “hegemonic paradigm” during the first two decades of the twenty-first century [34]. Covid-19 and viral neoliberalism must be understood as co-pathogeneses whereby the corona-virus pandemic has disclosed and reinforced global health insecurities due in large part to market-driven policies and neoliberal practices; the combination of societal inequalities, weakened response capacity by marketized health systems and biomedicine-oriented global health governance have created vectors of vulnerability [35].

The health security paradigm has its roots in concepts of risk mitigation and adaptation [3]. The Ebola outbreak in Western Africa in 2014–2015 has been important for the paradigm shift in global health policy. Since then, experts and politicians have been discussing the establishment of heath emergency funds, the formation of rapid reaction medical forces (‘white helmets’), as well as the creation of robust care structures and resilient health systems [36]. The 2015 Johannesburg Summit on China-Africa Cooperation identified Public Health as one of the cornerstones for foreign policy action in the China-Africa cooperation [37]. During their 2017 Berlin meeting the health ministers of the G20 countries simulated the necessary measures for combating future pandemics [38]. However, the prevailing reasoning does not aim to address the structural causes of potential threats [39], but serves as *“a form of immunization to have societies and systems become more resilient in dealing with future risks”* [40]. In this light, one should also analyse the process for establishing an international pandemic treaty, under WHO auspices, as a global normative framework for the preparedness and response to future pandemics [41]. This pandemic agreement, which at the time of writing is under negotiation by WHO member states, deserves critical scrutiny as its focus ought to be on people’s health, not merely national security. Creating genuine global health solidarity focused on human security requires a new pandemic agreement that mandates the sharing of technologies, scientific capacities and finance in future pandemics [42]. Wenham and colleagues argue that the key tension of the pandemic treaty is rooted in globalist ideals of what the perfect pandemic governance should look like, yet *“that it seems to have little regard for the realities of the statist, securitized landscape that exists for responding to pandemic threats… Even something as big as a major global pandemic is not sufficient to get governments to think beyond national interests”* [43].

## Blurred lines between hard and soft security practices

To a large extent, health security is viewed as a mainly biomedical challenge determined by biological dysfunctions and disturbances of the homeostatic state of the body. The guiding concepts for infectious disease control are based on the extended bacteriological model developed towards the end of the nineteenth century by the German physician and microbiologist Robert Koch, who discovered the specific causative cell-agents called bacteria, which cause fatal infectious diseases such as anthrax, tuberculosis, and cholera. Until today, despite all knowledge and empirical evidence about the enormous importance of non-biological influences on disease and health [44], the perception of health threats and the shape of health security are still dominated by a biomedical scientific approach derived from Koch’s “cellular pathology” [45]. Natural science medicine is considered to have made an important contribution to the increase in global life expectancy—even though social and environmental advances and the general improvement of living conditions worldwide have contributed to this in the first place—and is therefore considered to be a historically successful framework model for the prevention and treatment of infectious diseases [46]. Moreover, the biomedical understanding of health problems and the resulting importance of healthcare provision are plausible and relatively easy to explain and understandable for the general public [47, 48].

Notwithstanding this widespread perception, the biomedical concept is not conducive to adequately address the underlying conditions between public health status and disease outcomes. Already in the context of clinical conditions, biomedicine faces challenges vis-à-vis treating mental health problems or chronic degenerative diseases and their multifactorial causes that are prevalent worldwide today. Biochemical causal chains, organic defects or epigenetic variables and “markers” cannot be verified with certainty for numerous physical diseases and dysfunctions. Fundamental questions about the validity and relevance of the scientific biomedical paradigm therefore remain warranted [49]. This paradigm systematically neglects the consideration of non-medical variables and thus the social and ecological determination of health [50].

The dominant perception of health security as a defence against acute health threats openly reflected in high-level political statements about the “war on COVID-19” [51], is consistent with the (historical) involvement of defence forces in epidemic disease control [52]. In many countries, the military provided active and highly visible logistical support to the health sector in COVID-19 vaccination campaigns [53]. A few years ago, at the height of the Ebola crisis, “Médecins sans Frontières”, which otherwise rejects any proximity to the armed forces [54], demanded support from soldiers in controlling the epidemic and related social unrest [55]. Due to the increasing number and brutality of protracted armed conflicts and the growing need for humanitarian assistance, even NGO aid is increasingly becoming securitised with many organizations having to employ security personnel [56]. Ebola made it onto the agenda of the UN Security Council, and for the first time in the history of the UN, a mission to control a disease was established in the form of the “UN Mission for Ebola Emergency Response” [57]. While militarization is not the same as the securitization of health [58], the involvement of (armed) defence forces has contributed to the shaping of the global health security discourse. The military backing of state responses to national health threats has enforced the security narrative and thereby mobilised patriotic emotions of the general public which contradict the universality and shared responsibility required to foster global public goods for health [59, 60].

## Systemic shortcomings of global health security

Health security policies deal with future risks in such a way that they do not endanger the status quo of current political-economy inequalities and power relations. De Waal aptly notes that COVID-19 is not the nemesis of radical capitalism, but that rather “*the two parasitize on one another’s disruptive politics*” [61]. It is not the attention to the actual structural determinants of the pandemic that is at the centre of current political considerations, but rather the question of how to enable efficient crisis management [62]. The focus has been on how public health risks emerging from the animal world and environmental conditions can be identified and contained as early and directly as possible. The recent example has been the COVID-19 pandemic, which was governed by lock-down policies, a series of restrictions on socio-economic life and even curfews in many countries, as well as mass vaccination campaigns. Only few have raised the question of the upstream causes of the outbreak, amongst others the organization of food production in times of neoliberal globalization [63, 64]. The current global health security strategies and its financial mechanisms are not necessarily aimed at the protection of those who are most in need of social and human security—the poor and the marginalised. Instead, they pursue protecting the property, vested interests and privileges of the better-off [65]. Or to say it more bluntly; safeguarding the imperial way of life of some at the expense of many [10]. For overcoming the colonial and imperial heritage and addressing relevant health policy issues, global health needs to be decoupled from the global security approach, rather than deepening the connection [66]. This will require an extensive political process that must link a broader human security perspective to a systemic health-in-all approach that allows government efforts and investments to be regulated.

## Less biomedicine, more public health

Global health always contains a normative dimension, and global health practices and research, including on security matters, require normative premises that cannot be based solely on empirical evidence [67]. A moral language and deliberative process is requisite for ethical considerations that go beyond national, sovereign interests. At the same time, legal jurisdiction is needed for setting the rules of global health governance and remains best guided by human rights covenants [68]. The level of international negligence in the period between epidemic outbreaks is worrying. Long-term pandemic prevention, preparedness and health systems strengthening is needed in order to increase the security of people, societies and markets. However, governments, international organizations and global players mostly tend to neglect the underlying causes of epidemic risks and the unequal burden both caused and deepened by outbreaks. In fact, social movements in many places play a stronger role in sensitizing the public and fighting for health rights and entitlements than the state, although the latter is ultimately responsible for enforcing the right to health, including during epidemic crises [69].

It would be too simplistic to blame only biomedical researchers and their political advocates for a one-sided approach to the pandemic. The long neglect of the social determination of the impact of the COVID-19 pandemic is also attributable to the absence of a nuanced voice from the public and global health community, which tends rather to embrace the crisis frame because it brings the necessary attention and additional funds, and sometimes even adopts martial language itself [70]. Public health advocates subordinated themselves too long and too willingly to the fear-driven discourse that the media and politics readily reproduced [71–73]. Contrary to what could be expected vis-à-vis a veritable global health challenge, the pandemic response has only in a limited manner enhanced non-medical, social-science-oriented health research, and rather marginalised or even weakened it [7]. Simultaneously, due to their sheer financial power, philanthropic foundations such as the Gates foundation have enormous influence on academic and research agendas, health care supply and public policies worldwide [74]. Their focus on output-based performance measures and innovation further drives the verticalization of biomedical approaches at the expense of integration, interdisciplinary cooperation and wider system approaches [75, 76].

In order to develop and implement an appropriate and effective global health policy going beyond the current understanding of health security, much more than biomedicine, epidemiological surveillance, data integration or genetic-engineering is needed. Health security should primarily pursue the goal of all human beings to be protected from social, economic and ecological risks, while fulfilling health capabilities and equity, a concept known as ‘human security’ [77]. Overly securitised health policies have often prevented truly universal, public national health systems that promote health equity and envisage upstream determinants of health [78]. Global health security needs more focus on environmental health promotion, decent employment and income conditions for all, and other non-medical determinants of health. Health security policies must address the dubious influence of powerful transnational corporations, such as those involved in toxic waste processing, nuclear waste deposits, and hazardous chemical industries. Likewise, the responsibility of corporate powers on health security is evident in, among others, industrialised food production [79], as well as exploitative, unacceptable and impoverishing working conditions.

Global health policies must intervene beyond the health sector itself and address real and potential health threats and the socio-economic determination of health, including unpacking and pushing back the strong influence of corporate interests and the power they exert over health, mainly through their growing commodification and control of knowledge through the imposition of intellectual property rights [80]. Hence, regulating the power of transnational corporations should be a public good and particularly a global health priority as important as fighting epidemic threats [81]. This requires an intensive political and social struggle that goes beyond the health sector and health policies alone. Individual nation states and national governments are likely to be overwhelmed in addressing the health security challenges created by transnational corporations by regulating and taxing them more heavily. However, this is an urgent task for policy-makers, both to reduce health threats and to expand the financial scope for systemic improvements in health security [82].

While the necessary global governance structures are difficult to implement and enforce, health-in-all policies are ultimately required in order to integrate and articulate health considerations into policymaking across sectors [83]. The increasing emphasis on securitizing global health in the sense of combating acute and future health threats will, however, not make a significant contribution, if at all, to the basic conditions of people’s health, but rather tend to deteriorate them [84]. On the contrary, as Loewenson and colleagues have expressed in response to the securitization of the COVID-19 pandemic: *“experiences of comprehensive, equity-focused, participatory public health approaches, which use diverse sources of knowledge, disciplines and capabilities, show the type of public health approach that will be more effective to meet the twenty-first century challenges of pandemics, climate, food and energy crises, growing social inequality, conflict and other threats to health.”*[50].

The recent coronavirus pandemic has demonstrated the overarching and sorely neglected relevance of the political and, in particular, economic and commercial determination of health, and the need for them to be taken more seriously into account in all future investments, including ensuring the availability of global public goods such as vaccines and medicines [85]. Health security policies must ultimately focus on protecting the people of this world from the consequences of global economic inequalities as largely determined by neoliberal doctrine. Governments around the world have outdone each other by launching huge investment programmes at unprecedented levels in order to mitigate the immediate economic consequences of the COVID-19 outbreak [86, 87]. Likewise, the World Bank committed over 200 billion US dollars to public and private sector clients between April 2020 and March 2021 to overcome the economic and financial sequela of the pandemic [88]. During such a state of epidemic emergency, governments need to exercise the economic power vested in the state to address societal unrest and instability. As a response to several conflicts, food and economic crises, over 140 countries around the globe will impose new austerity measures on public expenditure in the upcoming post-pandemic years [89].

Such an imposition of executive power can provide fertile grounds for human-rights violations and may even facilitate further transformation from democratic-liberal to more authoritarian regimes [90]. History has taught us that emergency measures are often abused and maintained permanently. A growing number of governments has started to require citizens to install smartphone apps, allowing officials to track individuals and determine whether they can leave their homes. Without proper and legitimate governance this can lead to a form of surveillance capitalism [91].

De Waal suggests that we not merely have a pandemic crisis, but that we have a crisis in our way of life. He recommends to use the word ‘pandemy’ instead, as this better reclaims the concept of a holistic, socio-political, health and ecological pathology. As such, a response to address a ‘pandemy’ would need to integrate a comprehensive ‘One Health’ approach to identifying the root causes of disease with the ´People’s Science’ practice of responding to them [61].

## Conclusions

The fact that in recent years the global context of health has increasingly come to the fore in foreign policy is encouraging, as long as it is not reduced to epidemiological preparedness for preventing the cross-border spread of infectious diseases, particularly to high-income countries. Biomedical and technocratic reductionism leads to selective access to health care, and privatization increases rather than reduces health inequalities [50, 54]. Global health security policies have to take into account the complexity of health in its plurality and diversity; it can only be effective when it is recognised as a cross-cutting issue in all policy areas. Global health security must extend the concept of health threats beyond acute infectious diseases and ultimately apply to conditions and factors that have the potential to threaten people’s health. Health security must invariably envisage environmental, social, political, military, and commercial determinants [92] that put population health and equity under pressure and threaten people’s health and well-being, not only when a viral epidemic or pandemic arises. Thereby, health security must not disregard the structural causes of both communicable and non-communicable global health threats, inequities and impoverishment of many on this planet, namely the persistence of coloniality and its imperial mindset and practices [93] and a neoliberal capitalist economic order oriented towards short-term profit maximization and the ecological exploitation of natural resources in particular. Responsible global health security policy must address the underlying structures of existing problems and should not limit itself to merely sustaining the conditions that have led to the global and planetary health crisis to begin with.

## Data Availability

Not applicable.

## Abbreviations

COVID-19: Coronavirus disease 2019
G7: Group of 7
G20: Group of 20
NCD: Non-communicable disease
SARS-CoV-2: Severe acute respiratory syndrome coronavirus 2
UN: United Nations
WHO: World Health Organization
